# Data sharing in PLOS ONE: An analysis of Data Availability Statements

**DOI:** 10.1371/journal.pone.0194768

**Published:** 2018-05-02

**Authors:** Lisa M. Federer, Christopher W. Belter, Douglas J. Joubert, Alicia Livinski, Ya-Ling Lu, Lissa N. Snyders, Holly Thompson

**Affiliations:** NIH Library, Division of Library Services, Office of Research Services, National Institutes of Health, Bethesda, Maryland, United States of America; Tilburg University, NETHERLANDS

## Abstract

A number of publishers and funders, including PLOS, have recently adopted policies requiring researchers to share the data underlying their results and publications. Such policies help increase the reproducibility of the published literature, as well as make a larger body of data available for reuse and re-analysis. In this study, we evaluate the extent to which authors have complied with this policy by analyzing Data Availability Statements from 47,593 papers published in PLOS ONE between March 2014 (when the policy went into effect) and May 2016. Our analysis shows that compliance with the policy has increased, with a significant decline over time in papers that did not include a Data Availability Statement. However, only about 20% of statements indicate that data are deposited in a repository, which the PLOS policy states is the preferred method. More commonly, authors state that their data are in the paper itself or in the supplemental information, though it is unclear whether these data meet the level of sharing required in the PLOS policy. These findings suggest that additional review of Data Availability Statements or more stringent policies may be needed to increase data sharing.

## Introduction

In March 2014, PLOS announced a new data policy to promote public access to the research data underlying articles published in PLOS journals [1]. PLOS was among the first major publishers to enact such a policy, with many others adopting similar policies in the following years, including Springer Nature, Science, and Elsevier [2–4]. Publisher policies are part of a broader movement in scientific communities to encourage openness and sharing, along with funder mandates [5–7], statements from organizations and scholarly societies [8,9], and research on encouraging and facilitating sharing [10–12]. Such policies aim to facilitate validation and replication of research results, enable reuse of existing research data, and support data citation to ensure researchers receive credit when their data are reused. Though these policies all have similar goals, publishers vary in how strongly they word their policies, with some “encouraging” data sharing [4] while others “require” it. Since researchers are unlikely to share data in the absence of formal requirements or specific incentives, the distinction between encouraging and requiring can have significant implications for how many researchers actually make their data available [13,14]. Previous research that has shown journal requirements can have a significant impact in changing researchers’ behavior and increasing openness suggests that journal data sharing policies have the potential to meaningfully enhance data availability [15,16].

PLOS’s policy, like many other publishers’, requires a Data Availability Statement explaining how readers may access the relevant data, but “availability” can be interpreted in ways that have vastly different practical outcomes in terms of who can access the data and how. Ideally, the data underlying scientific research should be FAIR–findable, accessible, interoperable, and reusable–so that other researchers can locate and meaningfully use the data [9]. In practice, data are often difficult, if not impossible, to obtain if they are not made publicly available in a data repository. Researchers who have assessed the availability of data allegedly available upon request have had little success in obtaining data sets, whether because the original author could not be reached or refused to share, or the data were lost or unavailable [17–19]. To be effective in significantly increasing data availability, data sharing policies should prescribe mechanisms for sharing that ensure reliable and long-term access to data.

PLOS’s policy states that authors are required to “make all data underlying the findings described in their manuscript fully available without restriction, with rare exception” [20]. This policy calls for sharing of data beyond the type of summary data generally included in the body of a scientific paper, which would typically be inadequate for the type of reproducibility, reanalysis, and transparency that the full data set would enable. Though deposition of data in a public repository is “strongly recommended,” a number of methods for sharing data are also acceptable, such as including data as a supporting information file, making data available upon request through a data access or other approving committee, and providing contact information for a third party who owns the data [20]. Exceptions are granted if data cannot be made publicly available for legal or ethical reasons, such as when release could compromise patient or participant privacy or reveal the location of protected resources, like fossils or endangered species. However, data of a proprietary nature is not excepted from the policy, which notes that “if proprietary data are used and cannot be accessed by others (in the same manner by which the authors obtained it), the manuscript must include an analysis of public data that validates the conclusions so that others can reproduce the analysis and build on the findings” [20].

This study aims to explore how authors are sharing the data associated with their scientific articles by examining Data Availability Statements from articles published in PLOS. We have coded and analyzed the Data Availability Statements of nearly 50,000 papers published in PLOS between March 2014 and May 2016, using a combination of automatic and manual methods. This study complements and adds additional insights to the May 2017 blog post, in which PLOS reflected on the first three years of implementing their data sharing policy, estimating that about 20% of statements point to data in a repository, an equal amount are unavailable for privacy reasons, and the remaining 60% are available in the main text and supplementary information of the paper [21]. We consider authors’ data sharing practices at a more granular level than explored in the 2017 PLOS blog post, asking several research questions: 1) do most authors utilize PLOS’s “strongly recommended” sharing method of data deposition, 2) do authors utilize more than one method for sharing their data, 3) have sharing methods changed over time since policy implementation, and 4) among authors who have shared deposited data, what are the most popular repositories? By answering these questions, we hope to provide insight into how and where authors choose to share their data, how PLOS and other publishers may be able to further enhance data sharing, and how journals considering adopting a sharing policy might meaningfully increase data availability in their disciplines.

## Methods

We wrote a script to identify and retrieve relevant articles using the PLOS API, using R (version 3.1.1) and the rplos package [22]. This script retrieved 62,589 articles that met our inclusion criteria: 1) published in PLOS ONE, 2) publication date between March 1, 2014 (the date when the policy began) and May 31, 2016; and 3) article type of “Research Article”.

**Fig 1:**
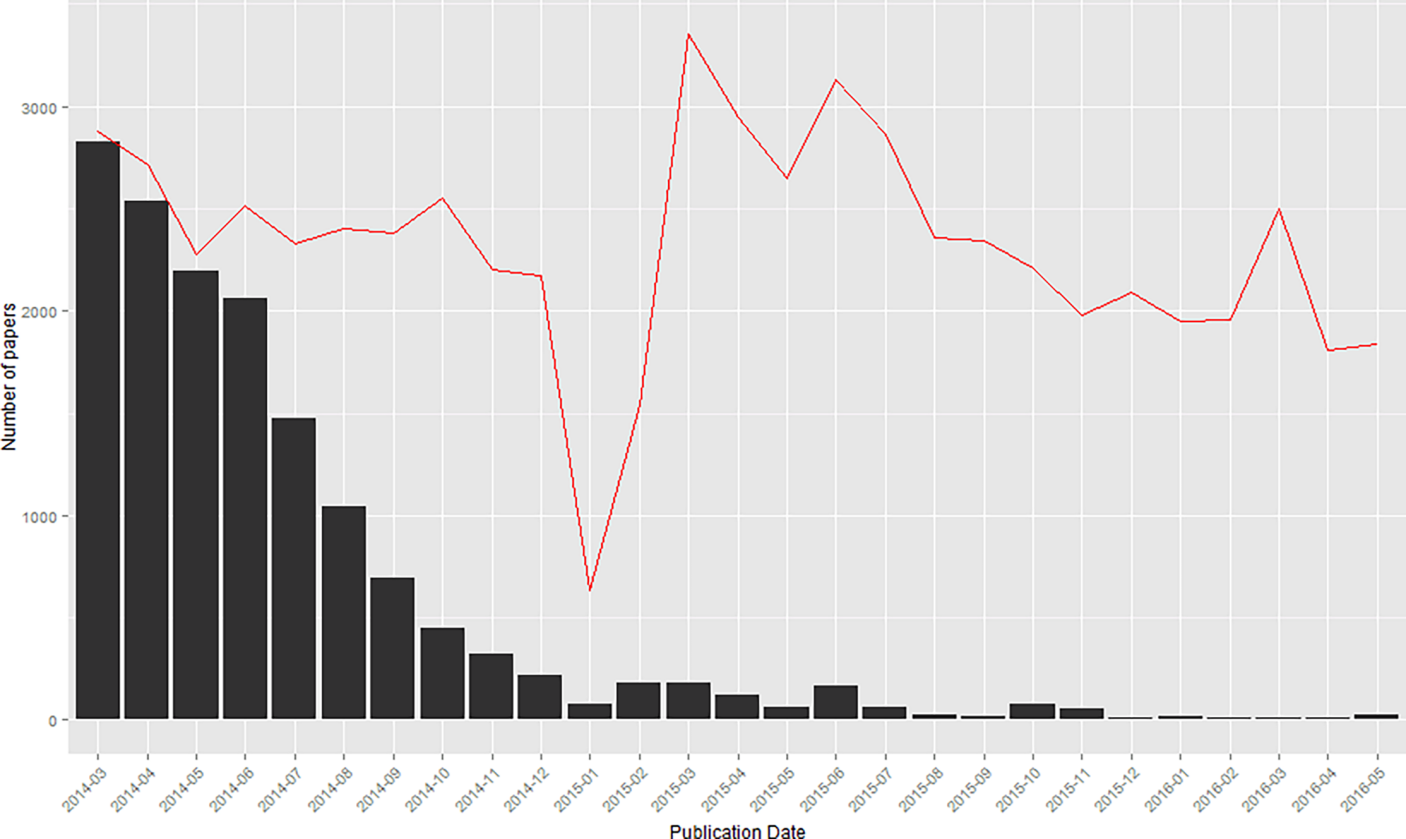
Articles missing a Data Availability Statement over time. The red line indicates total published articles, while the bars indicate articles with no Data Availability Statement.

Of the 62,589 articles that were retrieved, 14,928 (23.8%) did not contain a Data Availability Statement. As Fig 1 demonstrates, the majority of articles that did not contain a Data Availability Statement were published in the first few months following the start of the policy; these papers were likely accepted before the policy went into effect, even though they were published after the policy was implemented. In 2015 and 2016, the percent of papers that lacked a Data Availability Statement had dropped to 4% (n = 1041) and 0.7% (n = 73), respectively. It is unclear why papers were published without a Data Availability Statement after the policy was instituted.

**Fig 2:**
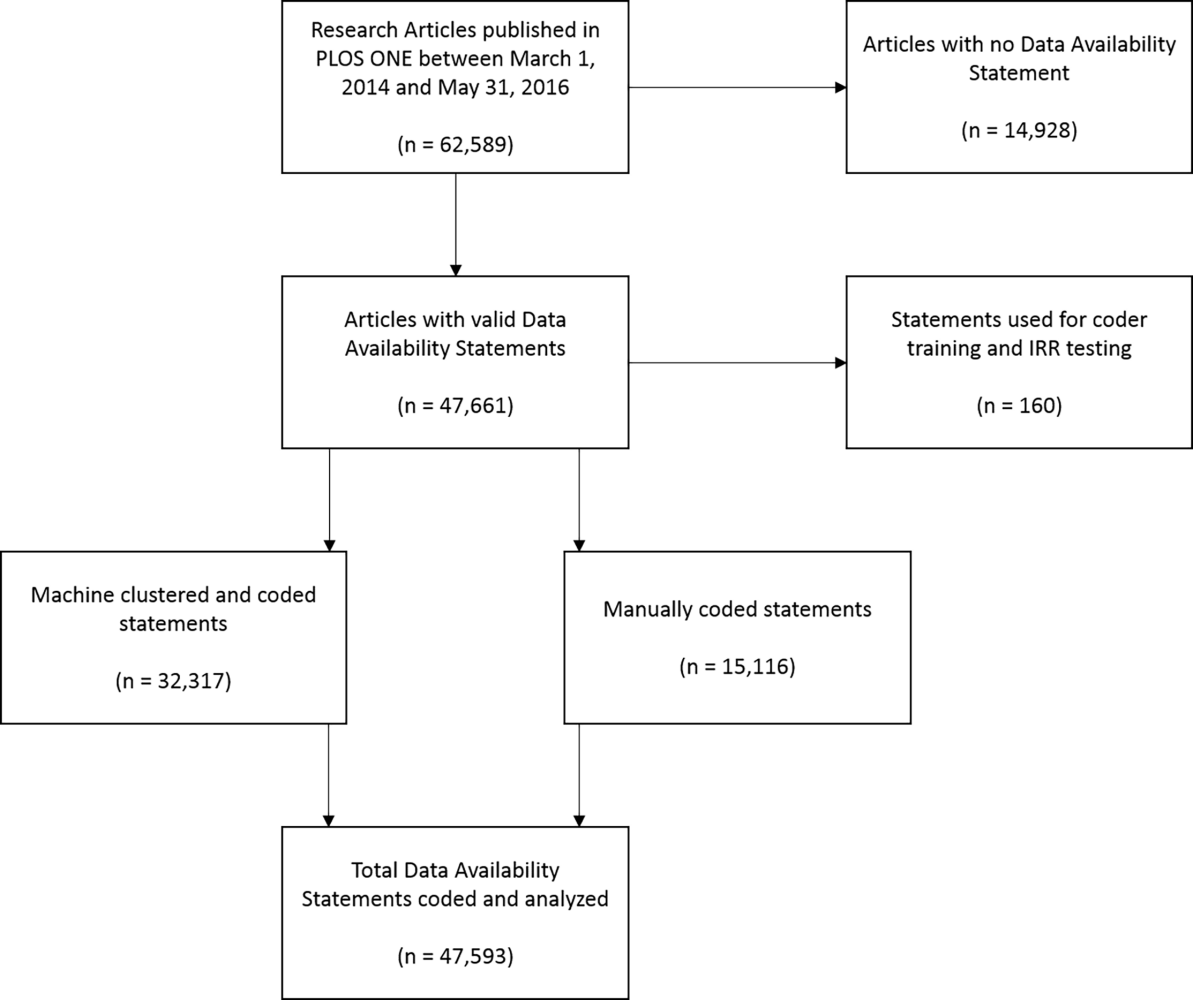
Flow diagram of Data Availability Statement coding.

Articles that did not contain a Data Availability Statement were removed, and the remaining 47,661 papers were used in this analysis. Where possible, these statements were coded automatically (as described below). Statements that could not be coded automatically were manually coded by the authors. A set of statements were randomly selected for inclusion in a coder training set and in a second set for assessing interrater reliability. Fig 2 demonstrates the flow of inclusion and coding.

### Automatic clustering and coding

Because many statements in the set were nearly identical, a large portion of the set could be coded automatically. For example, 15,128 papers used the identical statement “All relevant data are within the paper and its Supporting Information files.” A total of 288 text clusters were identified using the key collusion method with the cologne-phonetic and fingerprint keying functions in OpenRefine. These clusters were merged and labeled with a statement category according to the codebook (S1 Text. Codebook), resulting in 32,139 statements that had been coded. The remaining statements were sorted by count of occurrence and additional clusters of identical statements were manually identified. Sixty-two statements occurred 3 times or more in the data set, accounting for 338 total statements, which were coded accordingly. After coding all clusters, 32,477 statements had been categorized, leaving 15,116 unique statements that required manual coding.

### Manual coding

To ensure reliability across all raters, the research team met to discuss the draft codebook and complete a practice coding exercise on a subset of randomly selected statements, which were then removed from the final analysis. After this discussion, the codebook was revised and redistributed for use in a pilot test of statements randomly selected from the data set. The R packages irr [23] and psych [24] were used to calculate various measures of interrater reliability [25]. Fleiss’ kappa for *m* raters was 0.704 (z = 71.9, p = 0), considered Substantial agreement on Landis and Koch’s scale for Strength of Agreement [26]. Cohen’s kappa was also calculated pairwise for all raters; the mean of pairwise kappa scores was 0.701.

This level of reliability was agreed to be adequate, and after minor clarifying revisions, the final codebook (Appendix A) was distributed to the research team. The statements distributed to each coder also contained fifty statements that were common to all coders to allow for a final test of interrater reliability. For these fifty statements, Fleiss’s kappa for *m* raters was 0.81 (z = 51.2, p = 0) and the mean of pairwise Cohen’s kappa scores for all rater pairs was 0.82, both considered Near Perfect on the Strength of Agreement scale. After removing statements that had been used for coder training and in both phases of IRR testing, 15,116 unique statements had been manually coded. These were combined with the machine-clustered and coded statements, for a total of 47,593 statements to be analyzed.

## Results

Data Availability Statements were coded with one of ten statement categories. Table 1 describes the categories, contains a sample statement for each category, and indicates the number of statements coded within each category. The codebook (Appendix A) provides further detail about coding categories. Most statements indicate that the data were in both the paper and the supplemental information (45%) or in the paper alone (24%).

**Table 1:** Description of coding categories and example statements.

Code	Number of Statements	Definition	Sample Statement
access restricted	3,523 (7.4%)	statement mentions ethical, legal, or privacy restrictions, or the data are owned by a third party that restricts access	To protect potentially identifiable information on serious crimes, ethical approval is needed to access data. Data are available from <*source*> for researchers who meet the criteria for access to confidential data. For more information, see <*webpage*>.
combination	2,125 (4.5%)	statement mentions more than one mechanism for sharing	All data not contained within the paper or supporting files are available from <*repository*> (<*URL*>).
in paper	11,553 (24.3%)	statement indicates data are reported in the paper, including in tables and/or figures	The minimal data set underlying the findings in our study data is within the paper.
in paper and SI	21,568 (45.34%)	statement indicates data are reported in both paper and Supplemental Information	All relevant data are available from within the manuscript as well as a supplemental information file.
in SI	682 (1.4%)	statement indicates data are reported in the Supplemental Information	All data and analysis code have been provided as Supporting Information files.
location not stated	72 (0.2%)	statement says data are available but does not indicate where or how to locate the data	The authors confirm that all data underlying the findings are fully available without restriction. Data deposition.
N/A	17 (< 0.1%)	statement includes some boilerplate text but also adds N/A or Not Applicable	The authors confirm that all data underlying the findings are fully available without restriction. N/A.
other	31 (< 0.1%)	statement does not fit any of the nine categories	The authors confirm that all data underlying the findings are fully available without restriction. This paper is a theoretical discussion and therefore no data are involved.
repository	7,334 (15.4%)	statement names a publicly accessible location where the data are available, such as a repository or website	Data sets for all samples are available in <*repository*> under <*accession number*>.
upon request	688 (1.4%)	statement says that author or other individual or group must be contacted to access data	Data are available from <*name*>, who may be contacted by email at <*email address*>.

### Multiple mechanisms for sharing

A total of 2,125 statements (4.5%) mentioned more than one mechanism for sharing. For example, some statements noted that access to the dataset was restricted to protect study participants’ privacy, but that a partial dataset was available in a repository. The most common combinations were in a repository and in the paper and supplemental information (n = 951, 44%), in a repository and the paper (n = 313, 14.7%), and in a repository and supplemental information (n = 245, 11.5%).

### Trends in statements over time

**Fig 3:**
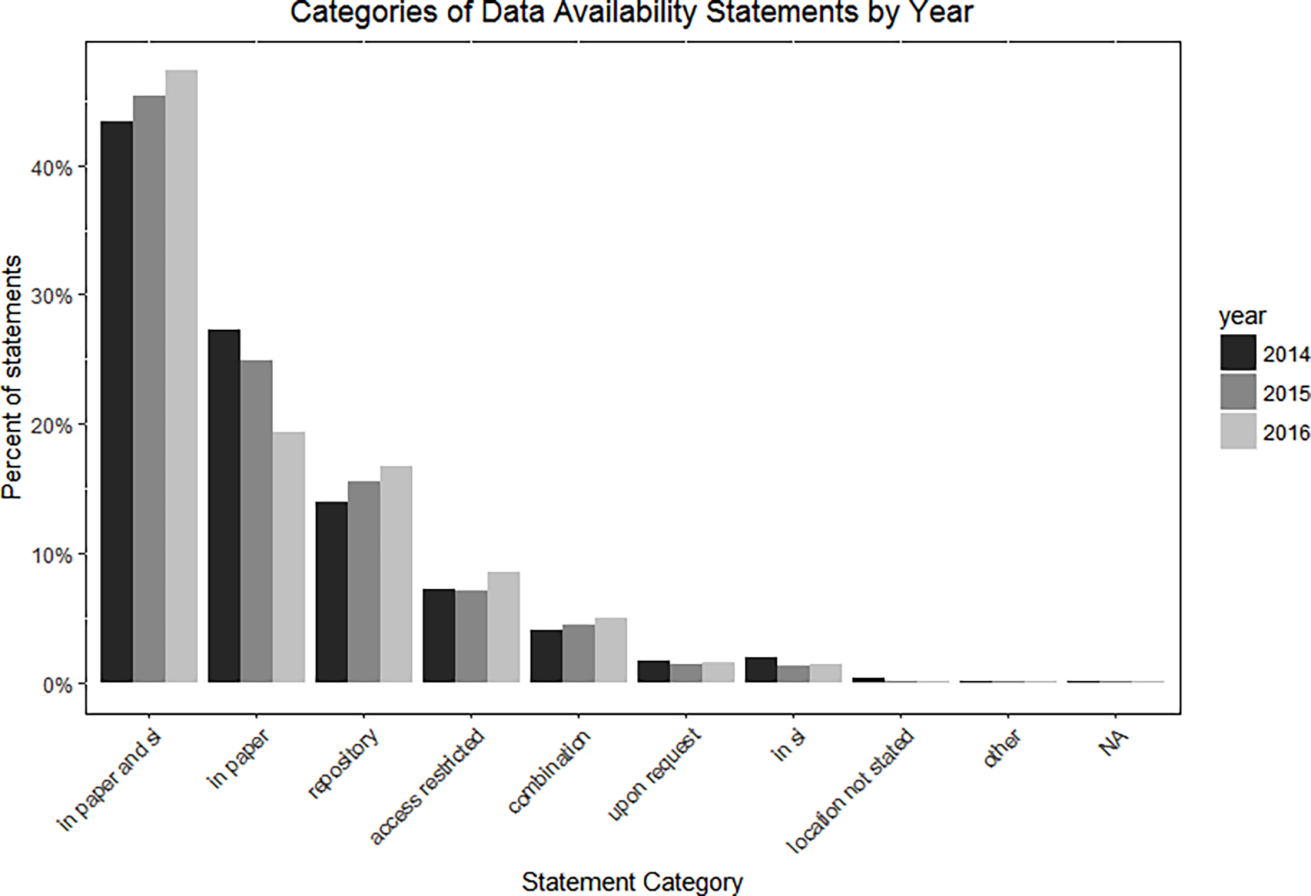
Distribution of statements across categories by year.

The distribution of statements across different categories remained mostly steady from the start of the policy to 2016, with slight increases over time in the number of statements indicating data were in a repository or in the paper and supplemental information, and a slight decrease over time in the number of statements indicating the data were in the paper. Fig 3 shows the distribution of statements across categories by year.

### Deposition in repositories

Our analysis identified 8,702 statements (18.2%) that named at least one specific repository or source where data were available (this number is greater than the number of statements coded with category “repository” because some statements coded as “combination” also mentioned a repository). Further, 974 unique repositories or sites were mentioned, and statements also mentioned sharing data in institutional repositories (n = 317) and non-repository websites (n = 329), such as a lab or researcher’s website. Of statements mentioning a repository, 7% mentioned more than one source, with six repositories being the most mentioned in a single statement. Table 2 shows the top twenty repositories by count of mention.

**Table 2:** Twenty most frequently mentioned repositories or sources.

Rank	Repository	Count of Mentions
1	Figshare	1,446
2	Gene Expression Omnibus (GEO)	1,001
3	Genbank	999
4	Dryad	987
5	Sequence Read Archive (SRA)	641
6	Non-repository website	329
7	Institutional repository	317
8	GitHub	280
9	Dataverse	217
10	Protein Databank (PDB)	172
11	National Center for Biotechnology Information (NCBI)	165
12	Open Science Framework	122
13	ArrayExpress	119
14	European Nucleotide Archive (ENA)	108
15	DNA Data Bank of Japan (DDBJ)	106
16	Zenodo	100
17	European Molecular Biology Laboratory (EMBL)	88
18	BioProject	79
19	dbGaP	64
20	Metagenomics Rapid Annotation using Subsystem Technology (MG-RAST)	45

## Discussion

Our findings, which were similar to those outlined in PLOS’s 2017 blog post [21], suggest that PLOS has had some success thus far in increasing the public availability of data associated with articles they publish. Nearly 20% of the articles analyzed in this study stated that their data were available in a public repository; by comparison, in a study conducted before PLOS’s policy went into effect, the investigators could only obtain one dataset out of ten even when they specifically requested them from the original authors [19]. These findings also suggest that PLOS’s policy may have had a greater impact on sharing than similar policies instituted by other journals. For example, study of articles published in BMJ between found that 63% of studies had statements indicating the data could not be shared, while another study found that only 24% of articles published in BMJ journals with a sharing policy had available data [27,28]. These differences in how BMJ’s and PLOS’s data availability statements affect actual data sharing practices may be due in part to the higher proportion of clinical trials published in BMJ, which may contain sensitive patient information that cannot be shared. Another study of randomized controlled trials published in *PLOS Medicine* and *BMJ* found that data could only be obtained for 46% of articles, suggesting the possibility that rates of sharing may in fact be lower among articles reporting on clinical trials [29]. That article did not identify subject privacy as a major factor in authors’ refusal to share, but a reason for refusal was only given in half of the studies for which data was not available. Future research investigating differences in data availability among journals with policies may yield further insights into what factors lead to increased data sharing.

Some research suggests that the data policy may actually discourage authors from choosing to publish with PLOS; two separate surveys of researchers found that about 25% of respondents reported they would avoid publishing in PLOS or other journals with data sharing policies [14,30]. As PLOS notes in their blog post, significant change is challenging, and their efforts are still “a work-in-progress” [21]. Our findings suggest potential areas for improving data availability and moving toward greater openness of scientific research data. These recommendations are not aimed only at PLOS, but are relevant to publishers, repositories, and other stakeholders interested in increasing data sharing.

First, peer review or editorial review of Data Availability Statements may help ensure that data are truly available to readers. In some cases, statements provided no information at all about how to locate the data, such as the ones coded “location not stated” or “N/A” in this study. Other statements suggested data were openly available, but failed to give enough information for readers to actually locate the data. For example, some statements provided accession numbers without indicating the repository or source that contained the data, such as “The nucleotide sequence data within the paper are available under the accession numbers <*accession numbers*>.” Other statements indicated a repository, but did not provide a URL, DOI, accession number, or other means by which readers would be able to locate the specific data within the repository. While these authors have apparently made an effort to make their data publicly available, in practice, their data are inaccessible to readers.

Other statements seem to have been entered as placeholders, potentially intended to be replaced upon publication of the article, such as “All raw data are available from the XXX [*sic*] database (accession number(s) XXX, XXX [*sic*])” or “The data and the full set of experimental instructions from this study can be found at <*repository name*>. [This link will be made publically [*sic*] accessible upon publication of this article.]” These two articles, published in 2016 and 2015, respectively, still contain this placeholder text as of this writing. These placeholders suggest a need for closer review by editors or reviewers to ensure that statements are appropriate and complete. While this type of review would require extra effort, incorporating checks as a part of the review process could help ensure that Data Availability Statements are clear and point readers to the correct data.

Our analysis also found inconsistencies in the ways that some authors cited repositories; some used abbreviations rather than the repository’s full name or used an incorrect name. For example, 388 statements referred to the National Center for Biotechnology Information’s (NCBI) “Sequence Read Archive” and others referred to it by its older name, “Short Read Archive,” while over 200 statements only used its abbreviation, SRA. Other statements seemed to conflate all of NCBI, indicating that they had deposited simply to “NCBI” or “the NCBI database,” when in fact NCBI administers many different databases. While a subject matter expert would possibly be able to discern the author’s intent, greater clarity and consistency would help readers more easily locate publicly available datasets. Review of Data Availability Statements could help increase uniformity, and repositories could also assist by providing suggested text for sharing to submitters. Having standard text for shared data could be helpful not only in the context of Data Availability Statements, but could also be placed on submitters’ websites and used in other contexts where data may need to be cited.

Although PLOS indicates in their policy that deposit of data in a public repository is “strongly recommended,” as their 2017 blog post and this study both found, the majority of authors do not utilize this sharing method. Instead, most authors state that the data are in the paper or in the supplementary information. Scientific articles often contain only summary data, not the entire dataset; these summary data may not be adequate for readers to reproduce the author’s findings, and have limited usefulness for reuse and reanalysis compared to the full, original dataset. The PLOS policy, along with other publisher and funder policies that mandate or encourage sharing, is a step toward increasing availability of scientific research data, but sharing represents a significant cultural shift for some research communities for whom using data repositories has not been the norm. PLOS’s list of suggested data repositories is a helpful resource for authors who are new to sharing and may be unaware of appropriate repositories [20]; similar efforts to help remove some of the burden on authors may encourage additional use of repositories.

PLOS’s policy also includes among its acceptable sharing mechanisms that data may be made available upon request. While some data are sensitive and access should indeed be restricted to authorized individuals who can demonstrate that they will adequately protect the data, such a restrictive mechanism for sharing is not necessary for most data sets. Besides restricting access, allowing authors to make data available upon request rather than requiring them to place data in a repository also increases the chances that data will be unavailable. Previous research has found that as little as 11% of data was readily made available upon request, with the likelihood of data being available rapidly declining over time [17–19,31]. The PLOS policy stipulates that a group, such as a data access or ethics committee, must be named as the request contact rather than an individual author, which could help mitigate data access problems resulting from the contact author being unreachable. Nonetheless, PLOS and other journals may wish to consider whether a policy of allowing data sharing upon request will adequately ensure availability of data, especially over time.

### Limitations

This study considers the content of Data Availability Statements only; we did not confirm whether the data could in fact be found in the location a statement specified. In other words, we did not verify whether data that were reported to be available in a repository or website could actually be found there nor whether data reported as being available upon request could be obtained from the individual or group noted in the statement. We also did not examine papers or their supplementary information to confirm whether they did indeed contain an adequate “minimal dataset” as defined by PLOS.

### Future research and policy directions

As more publishers, funders, and other stakeholders begin to require data sharing, researchers must adapt to these new requirements in communicating their science. Given the reluctance of many researchers to make their data publicly available, sharing requirements can play an important role in increasing transparency and reproducibility of science and creating a culture of broader data sharing across scientific communities. However, this study and others have demonstrated that researchers will not necessarily make their data fully available if doing so is only “recommended” or “encouraged,” so publishers may need to consider how to create policy and review procedures that do more to help increase the proportion of articles for which complete data sets are available.

Further research into how authors have responded to the PLOS requirement and other early policies could help elucidate some of the pain points that authors face in sharing and potentially inform institutions or libraries on what services they could provide to support their researchers in sharing. In addition, future research could consider whether the means by which data are currently shared are adequate for data reuse. For example, is the type of data included in a manuscript enough for a reader to reproduce the original results, or re-analyze the data for some other purpose? A better understanding of both the needs of readers and the issues faced by researchers in sharing data could help publishers and other stakeholders devise policies that are most likely to benefit the research community as a whole.

## Supporting information

S1 Text Codebook. The codebook contains categories used to code Data Availability Statements and their full definitions.
(DOCX)
