# Supplementary material for: Data sharing in PLOS ONE: An analysis of Data Availability Statements
Source: PLoS One. 2018 May 2;13(5):e0194768. doi: 10.1371/journal.pone.0194768 (PMC5931451; doi:10.1371/journal.pone.0194768)
Supplement: S1 Text — The codebook contains categories used to code Data Availability Statements and their full definitions. (DOCX) [file pone.0194768.s001.docx]

**S1 Codebook**

**Definitions**

**Data**: PLOS considers “minimal data set” that must be shared to be “the data set used to reach the conclusions drawn in the manuscript with related metadata and methods, and any additional data required to replicate the reported study findings in their entirety.” Because PLOS publishes papers from a wide variety of disciplines, the exact nature of the data associated with a manuscript may vary. Here, we define data as the digitally recorded documentation of research findings and their associated metadata, including:

- numeric and text data; images, audio, and video;
- databases or other collections of digital research objects;
- software, models, algorithms, and other code; and
- other digital materials necessary to understand and replicate research results.

Non-digital data, such as specimens or physical samples, are not included in our definition of data, nor are other types of research documentation not relevant to replication of the study, such as laboratory notes, correspondence or communications, and grant or ethics applications.

**Repository**: We define a repository as a publicly accessible, online source where data can be downloaded, retrieved, viewed, or accessed. Depending on the type of data and the research discipline, repositories may include subject-specific repositories (such as Gene Expression Omnibus for gene expression data), institutional repositories (such as a repository hosted by the researcher’s home university), or general repositories (such as Figshare or Dryad). Since data may also include code or software, code-sharing sites like GitHub and Zenodo are also included in this definition. We also include in this definition other publicly accessible online sources that provide access to data, such as a webpage hosted by the researcher or the laboratory.

| **Code** | **Description** |
| --- | --- |
| **access restricted** | statement mentions ethical, legal, or privacy restrictions, or the data are owned by a third party that restricts access. In other words, the authors are prevented from publicly sharing the data for some reason outside of their control.  Note: use this code whether or not statement notes that interested parties may apply for access to restricted data. |
| **location not stated** | statement says data are available but does not state where or how to locate the data  Note: use this code if the authors refer to future plans to place in a repository but do not state a specific repository |
| **upon request** | states that author or other individual or group must be contacted to access data. Also use this code when the statement indicates data are available in a closed repository (i.e., interested parties must apply for access to receive the data).  Note: if statement mentions some sort of legal, ethical, or privacy restriction, use code **access restricted** instead. If statement indicates that data are available in a public repository that requires registration only (but not application to receive data), use code **repository**. |
| **in paper** | data are reported in the paper/manuscript, including in tables and/or figures  note: also use this code if data are in another previously published paper |
| **in si** | data are reported in the Supplemental Information  note: also use this code if data are in the SI of another previously published paper |
| **in paper and si** | data are reported in both paper and supplemental information  Note: use this code when the statement indicates data are in paper and/or SI |
| **repository** | states a publicly accessible location where the data are available without having to make a request, such as a repository or website. If a public repository, record the name of the repository in the repository_name field (see repository abbreviations below).  Note: use this code for data that the authors have deposited themselves, as well as when the statement notes authors have reused data they got from a public repository. If statement indicates that data are in a repository, but interested parties must apply for access, use code **upon request** instead.  if the authors refer to future plans to place in a repository but do not state a specific repository, use code **location not stated** |
| **combination** | use when more than one mechanism is mentioned (for example, private data are available upon request, but a de-identified data set is available in a repository). Include the codes for all mentioned mechanisms in the Notes field, separated by commas (e.g., in paper, repository) |
| **other** | does not fit one of the above classifications |
| **N/A** | statement includes some boilerplate text but also adds N/A or Not Applicable |
